# Real-World Pharmacotherapy-Driven Cardiovascular Risk Prediction Using Interpretable Machine Learning and Jordanian EHR Data

**DOI:** 10.3390/medsci14030343

**Published:** 2026-06-24

**Authors:** Said Moshawih, Lobna Gharaibeh, Islam Alfreahat, Abeer Jabra Shnoudeh

**Affiliations:** 1 Department of Pharmaceutical Sciences, Faculty of Pharmacy, Al-Ahliyya Amman University, Amman 19111, Jordan; 2 Department of Pharmacy, Faculty of Pharmacy, Amman Arab University, Mubis 11953, Jordan; l.gharaibeh@aau.edu.jo; 3 Department of Biopharmaceutics and Clinical Pharmacy, Faculty of Pharmacy, Al-Ahliyya Amman University, Amman 19111, Jordan; i.alfreahat@ammanu.edu.jo (I.A.); a.shnoudeh@ammanu.edu.jo (A.J.S.)

**Keywords:** cardiovascular risk prediction, machine learning, electronic health records, JoRisk model, risk stratification

## Abstract

**Background:** Cardiovascular disease (CVD) remains the leading cause of mortality worldwide, with over 75% of deaths occurring in low- and middle-income countries, where conventional risk models often demonstrate poor calibration and limited generalizability. **Objective:** This study aimed to develop an interpretable, pharmacotherapy-informed machine learning model for cardiovascular risk prediction using national electronic health record (EHR) data from Jordan. **Methods:** A retrospective cohort study was conducted using approximately 600,000 individuals from the national Hakeem EHR system (2018–2022). Demographic, clinical, blood pressure, laboratory, and medication data were integrated to construct three datasets reflecting varying levels of feature completeness. Multiple machine learning models were benchmarked, followed by optimization, hybrid modeling, and probability calibration. Model interpretability was assessed using SHAP analysis. **Results:** The national cohort demonstrated a high cardiometabolic burden, with prevalence of hypertension (50.2%), hyperlipidemia (54.9%), and diabetes (47.9%). Antihypertensive and lipid-lowering therapies were more frequently used among CVD patients (56.9% and 49.6%, respectively). Treatment patterns were dominated by amlodipine (19.9%) and atorvastatin (74.4%). The final calibrated seed-bagged gradient boosting model achieved robust performance (ROC-AUC 0.844; PR-AUC 0.813) with consistent generalization across datasets. Key predictors included antihyperlipidemic therapy, systolic blood pressure variability, age, and sex. **Conclusions:** This study presents JoRisk, a calibrated and interpretable machine learning framework that integrates pharmacotherapy and clinical data for short-term cardiovascular risk prediction. The model demonstrates strong performance using routinely available EHR variables and offers a scalable decision-support tool for risk stratification in resource-constrained healthcare systems.

## 1. Introduction

Cardiovascular disease (CVD) remains the leading cause of death worldwide, accounting for approximately 18.6 million deaths annually and representing nearly one-third of global mortality according to the World Health Organization and the Global Burden of Disease (GBD) Study 2019 [1,2]. This epidemiological weight is compounded by its growing prevalence in low- and middle-income countries, where more than 75% of CVD-related deaths now occur due to transitions in diet, physical activity, and healthcare access [3]. The GBD analysis underscores a substantial rise in modifiable cardiometabolic risk factors, such as hypertension, dyslipidemia, obesity, and type 2 diabetes, as key drivers of CVD morbidity [1]. Beyond mortality, CVD also contributes to substantial years of life lost (YLL) and years lived with disability (YLD), escalating its societal and economic impact [4]. In this context, early identification of at-risk individuals and timely intervention are paramount. Effective preventive strategies not only attenuate the progression to overt cardiovascular events but also alleviate the escalating burden on healthcare systems globally [5,6]. This has galvanized interest in predictive modeling, artificial intelligence, and computational screening to identify high-risk individuals and subclinical disease in asymptomatic populations; such data-driven strategies have shown strong predictive capacity in biomedical research, including drug discovery and risk stratification [7,8]. Shifting from reactive to preventive cardiovascular care is therefore both a clinical necessity and a public health priority.

Conventional cardiovascular risk prediction tools such as the Framingham Risk Score, QRISK3, and the ACC/AHA Pooled Cohort Equations have long served as cornerstones in preventive cardiology, offering clinicians accessible and interpretable estimates of 10-year cardiovascular event risk [9,10,11]. They are widely adopted for their simplicity, reliance on routinely collected variables (age, sex, blood pressure, lipids, smoking status), and ease of integration into clinical workflows. However, their generalizability is increasingly questioned: most were developed in predominantly White, Western populations, limiting external validity in ethnically diverse or low- and middle-income settings, and their rigid variable sets prevent adaptation to population-specific risk profiles or emerging predictors. Calibration drift, a well-documented phenomenon whereby predictive accuracy deteriorates over time or across geographies, further undermines their utility, particularly in non-Western populations such as those in the Middle East or South Asia, where traditional scores have shown consistent under- or overestimation of risk [12,13,14].

The widespread adoption of electronic health records (EHRs) has made large-scale, longitudinal, real-world clinical data available for cardiovascular risk prediction across diverse populations [15]. Unlike traditional models, EHRs span structured and unstructured data, from serial vital signs and laboratory values to medication history and comorbidities, enabling richer phenotyping and temporally informed modeling. Machine learning (ML) approaches have emerged as powerful tools to harness this complexity, offering the ability to detect non-linear relationships, interactions, and time-dependent trends often missed by conventional regression models [16,17]. Recent studies have shown that ML algorithms, such as random forests and deep neural networks, can outperform traditional scores like Framingham and ACC/AHA equations in predictive discrimination and risk reclassification [18,19]. This synergy between EHRs and ML holds promise for developing more accurate, dynamic, and personalized cardiovascular risk stratification tools tailored to contemporary clinical environments.

Prior machine-learning studies have repeatedly shown modest but consistent gains over conventional equations when large routine-care datasets are used. Weng et al. trained several algorithms on 378,256 UK primary-care records and reported a best 10-year area under the curve of 0.745–0.764, exceeding the 0.728 obtained with the ACC/AHA equations [18]. Using 423,604 UK Biobank participants, Alaa et al. obtained an area under the curve of 0.774 with automated machine learning, compared with 0.724 for the Framingham score [20]. A recent systematic review of artificial intelligence cardiovascular risk models similarly concluded that machine learning approaches tend to improve discrimination and reclassification, while cautioning that external validation and calibration are frequently neglected [8]. These models, however, were almost exclusively developed in high-income Western populations and rarely incorporated longitudinal pharmacotherapy as a predictor. The present work was designed to address both gaps.

To our knowledge, this is the first nationally representative cardiovascular risk model developed on Jordanian EHR data, and one of very few in the Middle East to integrate dispensed medication exposure as a core predictor. We present a machine learning framework for predicting CVD risk using real-world data from Jordan’s national health system. By constructing multiple analytical cohorts with varying data completeness, we systematically evaluated and optimized several models to develop a robust, interpretable tool, exploring a range of algorithms and validation strategies. The resulting model demonstrated strong discrimination and reliable risk estimation, underscoring the feasibility of deploying ML-based CVD risk models in low- and middle-income settings, where traditional scores may underperform due to calibration drift and demographic mismatch.

## 2. Materials and Methods

### 2.1. Study Design and Data Source

We conducted a retrospective observational study using routinely collected data from Hakeem, Jordan’s national electronic health records (EHRs) platform maintained by the Ministry of Health. The study window was January 2018–December 2022. The master registry contained 600,000 unique patients after de-identification and deduplication. Institutional review board approval was obtained prior to data access; only de-identified data were analyzed.

### 2.2. Study Population and Diagnostic Cohorts

Diagnoses were ascertained from clinician-entered ICD codes in Hakeem during the study window. To ensure cohort homogeneity and temporal consistency, all patients with any record of CVD or related events prior to the baseline date were excluded from the analytical datasets. Only individuals free of prior CVD at study entry were followed longitudinally, and those who subsequently developed incident CVD during the observation period (January 2018–December 2022) were classified as new-onset CVD cases. This approach allowed us to distinguish between baseline risk prediction and post-baseline event occurrence while preserving accurate temporal relationships between exposures and outcomes.

We defined 11 a priori disease groups: history of CVD & familial risk factors; prevalent CVD; cerebral infarction; atrial fibrillation; diabetes mellitus; severe mental illness (SMI); systemic lupus erythematosus (SLE); rheumatoid arthritis (RA); depression; migraine; and chronic kidney disease (CKD). Disease groups and diagnosis criteria according to ICD codes for each group are specified in Supplementary Table S1.

From EHR tables, we extracted demographics (age, sex, marital status), diagnoses (per groups above), and laboratory tests: HbA1c, total cholesterol, LDL-C, HDL-C, and triglycerides (TG). Where multiple measurements existed, we computed per-patient baseline, mean (AVG), and standard deviation (STD) for HbA1c and systolic BP to reflect control and variability. Implausible values (unit mismatches, non-physiologic ranges) were screened and set to missing.

### 2.3. Measurement-Based Identification of Cardiometabolic Conditions Using Guideline Thresholds

To report condition prevalence from measurements (separate from ICD labels), we used contemporary guideline thresholds and required confirmation (≥2 abnormal readings on distinct dates) where available:

- Hypertension: office SBP ≥ 130 mmHg or DBP ≥ 80 mmHg (ACC/AHA 2017), confirmed on ≥2 readings [21].
- Diabetes (HbA1c): HbA1c ≥ 6.5% on two tests, or one abnormal plus a confirmatory abnormal by another ADA-accepted test (per ADA Standards) [22].
- Hypercholesterolemia/dyslipidemia: LDL-C ≥ 130 mg/dL and/or Total-C ≥ 200 mg/dL (AHA/ACC lipid guidance); Hypertriglyceridemia: TG ≥ 150 mg/dL; low HDL-C per sex where available (if sex missing, <40 mg/dL used conservatively). Values required ≥2 abnormal results when longitudinal data permitted [23].

These thresholds were used strictly to compute measurement-based prevalence; ICD cohorts were analyzed separately as diagnostic groups.

### 2.4. Medication Exposure

Medication exposure was assessed from dispensed prescriptions in the Hakeem system, focusing on chronic oral therapies. Five major pharmacological classes were included in this evaluation: antihypertensive agents, lipid-lowering medications, corticosteroids, atypical antipsychotics, and phosphodiesterase-5 (PDE5) inhibitors. Only oral formulations (tablets and capsules) were eligible, ensuring consistent therapeutic intent and route. Prescriptions were excluded if non-oral (e.g., injectables, inhalers, topicals), lacking essential metadata, or reflecting minimal use (<30 tablets dispensed over the study period), to avoid incidental or short-term treatments. The comprehensive list of analyzed drugs is provided in Supplementary Table S2. Medication usage statistics presented in the Section 3 reflect within-class proportional consumption based on total dispensed volume across the Hakeem database, rather than individual patient-level prevalence, unless explicitly stated.

The five pharmacological classes were selected a priori on the basis of documented mechanistic or epidemiological links to cardiovascular risk. Antihypertensive and lipid-lowering agents are direct markers of treated hypertension and dyslipidaemia, the two dominant modifiable risk factors in this cohort [21,23]. Long-term corticosteroid exposure is associated with hypertension, dyslipidaemia and incident cardiovascular events [24]. Atypical antipsychotics carry well-characterised cardiometabolic liabilities, including weight gain, dyslipidaemia and QT prolongation, and were therefore retained as a risk-relevant exposure [25]. Phosphodiesterase-5 inhibitors were included because they are predominantly prescribed for erectile dysfunction, itself an early sentinel of endothelial dysfunction and subclinical atherosclerosis [26]. Drug groups without a consistent, routinely dispensed cardiovascular signal were deliberately excluded to preserve model parsimony and interpretability.

Importantly, these medication classes were used not only as exposure variables but also as a more reliable means of ascertaining the underlying medical conditions. Because the clinician-entered diagnostic codes in the Hakeem system are known to be incompletely and inconsistently recorded, we verified the presence of several conditions through dispensed medication records rather than relying on diagnostic codes alone. As the Ministry of Health dispenses these chronic therapies only when the corresponding medical condition is documented, the dispensing of a given drug class serves as a high-specificity proxy for the associated diagnosis, for example, antihypertensive dispensing for treated hypertension, lipid-lowering dispensing for dyslipidaemia, corticosteroid dispensing for chronic inflammatory conditions, and phosphodiesterase-5-inhibitor dispensing for erectile dysfunction. This medication-anchored ascertainment improves diagnostic accuracy within the dataset despite the limitations of the underlying electronic-health-record coding, and it explains why a condition such as erectile dysfunction is represented in the model through a medication-confirmed condition flag rather than as a separate pharmacological feature.

### 2.5. Data Preprocessing and Quality Control

All tables were indexed by PATIENT_ID and de-duplicated. Units were harmonized (mg/dL for lipids; % for HbA1c; mmHg for BP), and a common pipeline was applied across datasets: median imputation of missing continuous features and z-score standardization. We built three analysis datasets to handle missingness and to test model robustness as in Table 1.

Three datasets were constructed to make explicit the trade-off between feature completeness and cohort representativeness that characterises real-world electronic health record data. Dataset 1 retained every patient with a complete laboratory profile (HbA1c and a full lipid panel) and therefore preserved the true population event rate, but at this completeness threshold only about 5% of eligible patients had developed incident cardiovascular disease, yielding a severely imbalanced cohort of 10,130 records containing 517 positive cases. Dataset 2 matched these 517 positive cases one-to-one with laboratory-complete controls to create a balanced but small cohort of 1034 records, isolating model behaviour from prevalence effects. Dataset 3 relaxed the laboratory-completeness requirement and retained the routinely captured predictors (age, sex, systolic blood pressure measures, comorbidities and medication exposure); this allowed many more confirmed positive cases to qualify, producing a larger balanced cohort of 4672 records (2336 positive and 2336 negative). Class imbalance in Dataset 1 was addressed through synthetic minority oversampling and positive-class weighting, whereas Datasets 2 and 3 were balanced by design. Dataset 3 was pre-specified as the basis for the final deployed model because it offered the most favourable balance of sample size, outcome balance and feature availability.

Harmonisation was performed before any modelling. Laboratory results recorded in mmol/L were converted to mg/dL using standard analyte-specific factors; HbA1c was standardised to percentage units; and blood pressure was expressed in mmHg. Records were deduplicated for PATIENT_ID, and repeated measurements for each patient were consolidated into baseline, mean and standard deviation summaries for HbA1c and systolic blood pressure. Physiologically implausible values (for example, HbA1c above 20%, or blood pressure entries arising from unit mismatch) were screened and set to missing, after which median imputation and z-score standardisation were applied within cross-validation folds to prevent information leakage.

### 2.6. Modeling Framework

All analyses were conducted in Python 3.11 using scikit-learn, imbalanced-learn, TensorFlow/Keras and Optuna. We first benchmarked a broad set of classifiers, including logistic regression, k-nearest neighbours, support vector classifiers with linear and radial basis function kernels, decision tree, random forest, AdaBoost, gradient boosting and XGBoost. Models were implemented within a unified preprocessing pipeline adapted from consensus-based high-dimensional biomedical modelling workflows [27]. The pipeline included median imputation of missing continuous variables, z-score standardisation, optional synthetic minority oversampling using SMOTE, and optional principal component analysis for dimensionality reduction [28]. Unless otherwise specified, data were split into stratified training and testing sets using an 80:20 ratio. Model selection used GridSearchCV with five-fold stratified cross-validation, with precision–recall area under the curve (PR-AUC) as the primary optimisation metric because of class imbalance. Accuracy, precision, recall, F1-score, ROC-AUC, PR-AUC and Matthews correlation coefficient (MCC) were additionally reported.

For dataset-specific performance optimisation, the best-performing tree-based learner, Gradient Boosting or AdaBoost, was further tuned using Bayesian optimisation in Optuna [29]. Preprocessing was embedded within scikit-learn pipelines to prevent information leakage during cross-validation [30]. The Optuna objective combined classification balance and discrimination:

$$J_{\mathrm{boost}}=0.70\times F1+0.20\times ROC\text{-}AUC+0.10\times PR\text{-}AUC$$

Each configuration was assessed by five-fold stratified cross-validation, and 80 trials were performed per dataset. The selected model was then retrained on the full training set and evaluated on the held-out test set using ROC and precision–recall curves, confusion matrices and calibration plots generated with Matplotlib 3.10.8 and Seaborn 0.13.2.

To assess whether nonlinear representation learning could improve performance, we built a hybrid ensemble combining the optimised tree-based model with a deep neural network: a fully connected multilayer perceptron with three hidden layers (128, 64, 32 neurons). Hidden layers used ReLU activation, batch normalisation and 0.3 dropout [31]. The sigmoid output estimated CVD risk probability. Training used the Adam optimiser (learning rate 0.001), binary cross-entropy loss and early stopping (patience eight epochs) [32]. Final hybrid probabilities were obtained by weighted averaging:

$$P_{Hybrid}=w_{DNN}\times P_{DNN}+(1-w_{DNN})\times P_{base}$$

The neural network blending weight $w_{\mathrm{DNN}}$ was optimised over 40 Optuna trials with F1-score as objective; binary predictions used a fixed 0.5 threshold, with performance assessed by five-fold cross-validation (ROC-AUC, PR-AUC, F1, calibration plots, confusion matrices).

Finally, to improve probability reliability and reduce initialisation variance, we implemented a calibrated seed-bagged boosting ensemble: five boosting models with identical hyperparameters but different random seeds, each fitted within the same pipeline (median imputation, z-score standardisation, class weighting where applicable). Because boosting algorithms may produce poorly calibrated probabilities, each base learner was post hoc calibrated using isotonic regression via CalibratedClassifierCV with five-fold cross-validation [30,33]. The final ensemble probability was computed as the mean calibrated probability across the five seed-specific models. Two ensemble-level parameters were optimised with Optuna: the positive-class weighting factor, pos_weight∈[0.5,3.0], and the classification threshold, τ∈[0.20,0.90]. The optimisation objective was:

$$J_{CSB-Boost}=0.55\times F1+0.25\times MCC+0.10\times ROC\text{-}AUC+0.10\times PR\text{-}AUC$$

Each trial was evaluated by five-fold stratified cross-validation. After convergence, all five calibrated ensemble members were retrained on the full training set using the selected pos_weight and threshold values, typically approximately 1.6 and 0.45, respectively. Final evaluation was performed on the held-out test set and confirmed by additional stratified cross-validation using F1-score, MCC, ROC-AUC, PR-AUC and reliability curves.

For the final Dataset 3 model, the 4672 records were partitioned using a stratified 80:20 split with a fixed random seed of 42, yielding 3737 patients for training and 935 patients for held-out testing; class proportions were preserved in both partitions.

Three analyses were added to strengthen clinical interpretation and to align with established reporting standards. First, to quantify added value over routinely available inputs, a conventional logistic risk-factor model using only age, sex and baseline systolic blood pressure was trained and evaluated on the identical training and test partitions, and the difference in area under the curve was assessed with a paired bootstrap of 2000 resamples. Second, decision curve analysis was used to estimate net clinical benefit across a range of decision thresholds, comparing the model with treat-all and treat-none strategies [34]. Third, an algorithmic-fairness audit was performed by stratifying discrimination, calibration and error rates by sex, in line with recommendations on bias in clinical prediction algorithms [35]. A literal application of the Framingham and QRISK3 equations was not feasible on this cohort because several of their required inputs (smoking status, body mass index, complete cholesterol panels for all patients, and ethnicity or deprivation indices for QRISK3) were not uniformly recorded; the conventional age/sex/blood pressure model therefore served as the same-data comparator, with runnable code provided for the full equation comparison once these covariates are linked.

**Figure 1:**
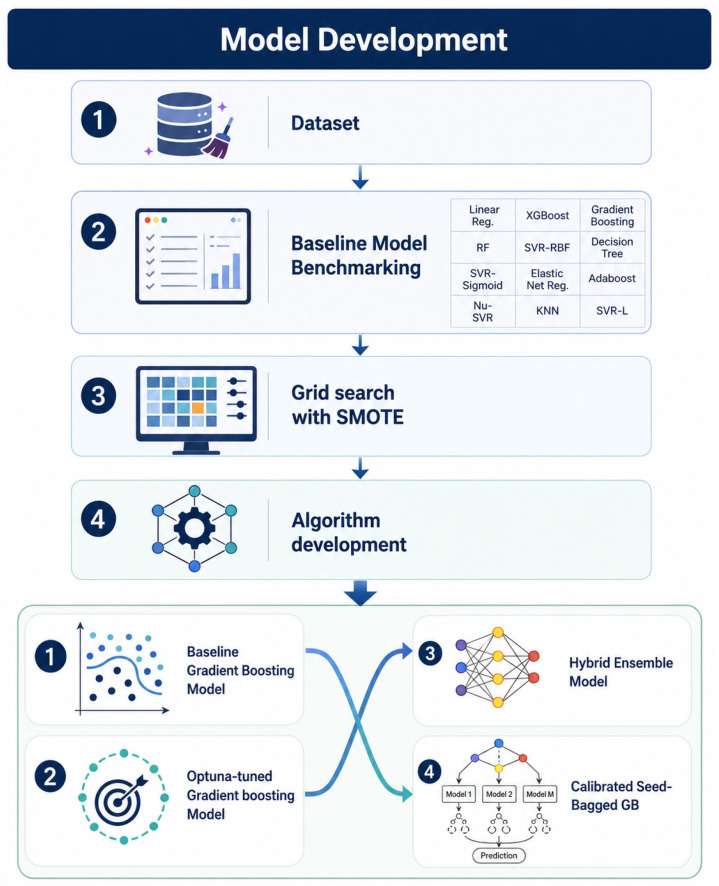
Schematic workflow of model development and optimization from the different Datasets, illustrating the progression from baseline model benchmarking and grid search with SMOTE to advanced Gradient Boosting-based optimization, including Optuna tuning, hybrid ensemble integration, and calibrated seed-bagged refinement.

Finally, to quantify in-sample optimism and provide a formal assessment of overfitting beyond the held-out partition, we applied Harrell’s optimism correction bootstrap [36,37], as recommended by the TRIPOD guidance for prediction models [38]. The complete modelling procedure, including imputation, standardisation, isotonic calibration and seed-bagging, was refit on each of B = 200 bootstrap resamples; for every resample the difference between performance on the bootstrap sample and performance on the original full sample was computed, and the average of these differences provided the estimated optimism. Optimism was then subtracted from the apparent (full-sample) performance to yield optimism-corrected estimates of discrimination (ROC-AUC, PR-AUC) and calibration (Brier score). The final structure of methods followed was summarized in Figure 1.

## 3. Results

We analyzed a national EHR cohort of approximately 600,000 individuals in Jordan’s Hakeem system to characterize the burden of CVD, major cardiometabolic comorbidities, and medication-utilization patterns. Prevalence estimates were derived from longitudinal clinical and laboratory data using internationally standardized criteria. Table 2 presents the distribution of categorical variables stratified by CVD status, highlighting demographic and clinical distinctions between individuals with and without cardiovascular events. Notably, females constituted a slight majority of the cohort, yet the prevalence of CVD was significantly higher among males (59.8%) compared to females (40.2%, *p* < 0.001), aligning with global epidemiological patterns. Age was a strong determinant of CVD risk: 46.2% of CVD cases occurred in individuals older than 60 years, whereas only 13.0% were younger than 40, with highly significant differences across age strata (*p* < 0.001).

The dataset is representative of an older population with relatively low comorbidity burden. Most participants had no documented history of prior CVD, and conditions such as atrial fibrillation, chronic kidney disease, and schizophrenia were uncommon but showed significant enrichment among CVD patients. For example, atrial fibrillation was present in 1.76% of CVD cases compared to 0.34% in non-CVD (*p* < 0.001), while chronic kidney disease showed an even starker difference (2.95% vs. 0.90%, *p* < 0.001). Rheumatologic disorders such as rheumatoid disease and lupus were infrequent and showed no statistically significant association with CVD outcomes, potentially due to limited representation or underdiagnosis in the dataset.

Regarding medication exposure, antihypertensive and lipid-lowering therapies were the most frequently used chronic medications across the cohort. Among CVD patients, 56.9% had been prescribed antihypertensives and 49.6% received antihyperlipidemic agents, both significantly higher than the proportions in the non-CVD group (48.3% and 47.3%, respectively; both *p* < 0.001). Corticosteroid and antipsychotic use also demonstrated significant differences, indicating their potential indirect association with cardiovascular outcomes. These trends reflect the higher therapeutic burden among individuals with CVD.

**Figure 2:**
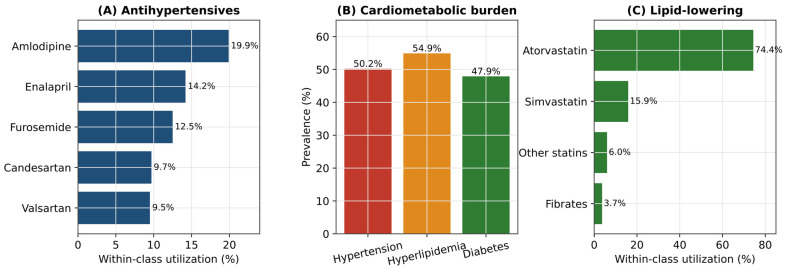
Cardiometabolic Disease Burden and Treatment Patterns in Jordan’s Public Healthcare Sector. Prevalence of hypertension, hyperlipidemia, and diabetes (**B**) and distribution of commonly prescribed antihypertensive (**A**) and lipid-lowering therapies (**C**) based on real-world data from the national Hakeem system.

Hypertension, hyperlipidemia, and diabetes prevalence were determined in accordance with the American Heart Association (AHA, 2017) [21] and American Diabetes Association (ADA, 2023) [22] guidelines. Hypertension was identified based on systolic blood pressure (SBP) ≥ 130 mmHg or diastolic blood pressure (DBP) ≥ 80 mmHg on at least two separate encounters. Hyperlipidemia and hypercholesterolemia were defined using serum lipid measurements consistent with AHA thresholds (LDL-C ≥ 130 mg/dL, triglycerides ≥ 150 mg/dL, and/or total cholesterol ≥ 200 mg/dL), confirmed on at least two occasions. Diabetes classification followed ADA diagnostic criteria based on laboratory HbA1c values. Using these standardized thresholds, the prevalences of hypertension, hyperlipidemia, and diabetes across the national cohort was 50.2%, 54.9%, and 47.9%, respectively in Figure 2 (Panel B). These rates reflect the proportion of patients with sufficient clinical data available within the Hakeem system to meet the diagnostic criteria over the study period, representing real-world disease burden within Jordan’s public health network.

Panels A–C in Figure 2 summarize the distribution of cardiometabolic pharmacotherapy utilization derived from prescription records collected from government hospitals and government primary healthcare centers during the same observation period within the national Hakeem electronic health record system. Antihypertensive therapy patterns (Panel A) demonstrated predominant use of calcium channel blockers (e.g., amlodipine, 19.9%) and renin–angiotensin system blockers (e.g., enalapril, 14.2%), followed by diuretics (e.g., furosemide, 12.5%) and angiotensin-II receptor blockers (e.g., candesartan 9.7%; valsartan 9.5%). The lipid-lowering medication landscape (Panel C) was characterized by dominant statin therapy, with atorvastatin comprising 74.4% of prescriptions, followed by simvastatin (15.9%), while fibrates accounted for a smaller proportion of use. These medication patterns represent relative class-specific utilization rather than absolute prescription volume, reflecting therapeutic preference and clinical practice trends across the national healthcare system.

### 3.1. Performance Benchmarking and Model Advancement for Dataset-1

Benchmarking identified the most suitable baseline classifier for Dataset-1. Among nine models, the Linear SVC showed the most balanced, generalizable performance. With optimized regularization (C = 0.1) and dimensionality reduction to 15 principal components, the Linear SVC achieved the highest precision–recall performance (PR-AUC = 0.143; Cross-Validation CV PR-AUC = 0.122), indicating enhanced stability in distinguishing true CVD cases from non-cases. Logistic Regression yielded comparable PR-AUC (0.142) but lower global discrimination, whereas tree-based models such as Gradient Boosting and Random Forest showed moderate sensitivity with reduced precision. In contrast, XGBoost exhibited exceedingly high recall (0.97) coupled with very low precision (0.06), reflecting an over-aggressive response to class imbalance and limited clinical utility. Based on these findings, the Linear SVC was designated as the optimal baseline for subsequent optimization (Supplementary Table S3).

Optuna tuning refined the Linear SVC over the penalty parameter C, yielding an optimal value of C = 0.0638, which produced an accuracy of 0.950, precision of 1.000, and recall of 0.019 (F1-score = 0.038). Although recall remained low due to severe class imbalance, the tuned model maintained stable discrimination (ROC-AUC = 0.596; PR-AUC = 0.090) and strong reproducibility across CV data (CV ROC-AUC = 0.634; CV PR-AUC = 0.121), confirming resistance to overfitting. Re-evaluation with repeated cross-validation validated full reproducibility of these metrics (MCC = 0.136), reinforcing model stability across random seeds.

Throughout this work, “unseen” denotes the held-out validation partition that was never used during training or hyperparameter selection; cross-validation estimates and held-out estimates are reported separately. It should also be emphasised that the low precision–recall performance on Dataset 1 is an expected consequence of its approximately 5% event prevalence rather than a failure of the modelling approach: at this level of imbalance precision is bounded by the base rate, and the area under the curve values reported here are consistent with those obtained by large population-scale studies on comparably imbalanced cohorts [18,20]. Dataset 1 was retained only to characterise behaviour under realistic prevalence and was not the basis for the deployed model.

**Figure 3:**
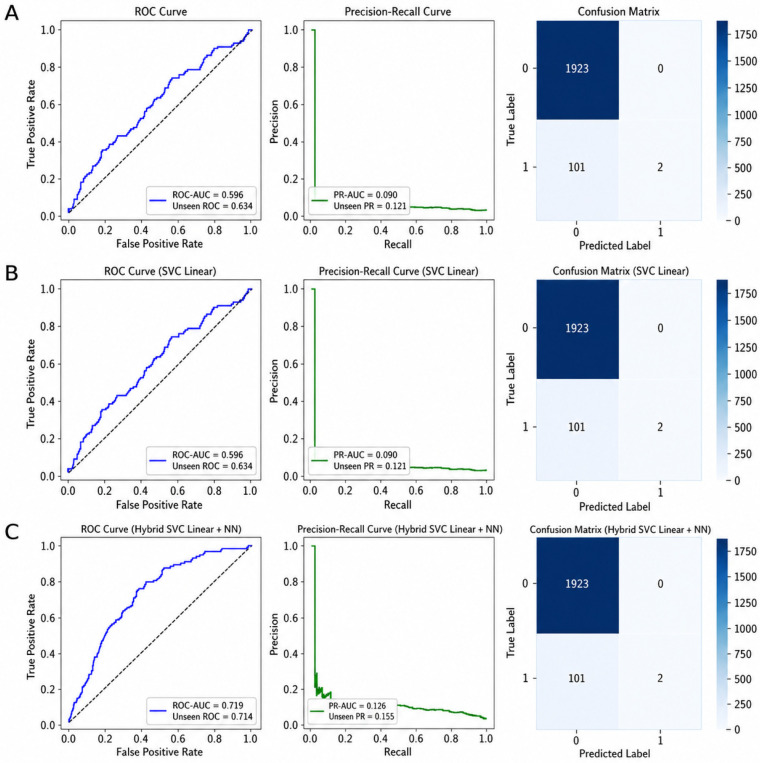
Performance comparison of models on Dataset-1. ROC, precision–recall curves, and confusion matrices for (**A**) baseline SVC, (**B**) tuned SVC, and (**C**) hybrid SVC-NN model, showing progressive improvement in discrimination and stability.

To further enhance representation capacity, a hybrid SVC–Neural Network (NN) ensemble was constructed by combining the tuned SVC with a feed-forward deep neural network. Optuna-guided blending identified an optimal neural network contribution weight of w_NN_ = 0.35, with the remainder attributed to the linear SVC decision function. The ensemble preserved accuracy and precision (Accuracy = 0.950, Precision = 1.000) but yielded notable gains in discrimination: ROC-AUC increased to 0.719 and PR-AUC to 0.126, with CV performance also superior (CV ROC-AUC = 0.714; CV PR-AUC = 0.155). This shows neural representation learning can enhance ranking and sensitivity on highly imbalanced data without compromising stability. The progression from baseline SVC to tuned SVC to hybrid ensemble is shown in Figure 3A–C (ROC, precision–recall curves, confusion matrices), with all model values in Supplementary Table S4.

### 3.2. Model Benchmarking and Incremental Optimization for Dataset-2

For Dataset-2 (approximately 1000 patients), benchmarking determined the best baseline classifier prior to optimization [27]. Ensemble learning methods consistently outperformed linear and distance-based models, demonstrating superior discrimination and minority-class sensitivity. Among all algorithms evaluated, AdaBoost achieved the strongest baseline performance, yielding the highest PR-AUC (0.714) and F1-score (0.673), indicating a favorable precision–recall trade-off and effective detection of cardiovascular events. Random Forest and Gradient Boosting models demonstrated stable but slightly lower performance, while the Decision Tree baseline showed limited predictive capability, underscoring the advantage of boosting-based aggregation for cardiovascular risk prediction in this dataset. Accordingly, AdaBoost was selected as the baseline model for further optimization as illustrated in the Supplementary Table S5.

Optuna tuning of AdaBoost improved discrimination (test F1 = 0.651, ROC-AUC = 0.723, PR-AUC = 0.709), with further gains on unseen data (ROC-AUC = 0.740; PR-AUC = 0.713), confirming minimal overfitting. Re-evaluating the fixed best hyperparameters across independent splits gave consistent performance (test F1 = 0.647; ROC-AUC = 0.725) with improvement on unseen data (ROC-AUC = 0.748; PR-AUC = 0.731), confirming stability of the tuned configuration.

**Figure 4:**
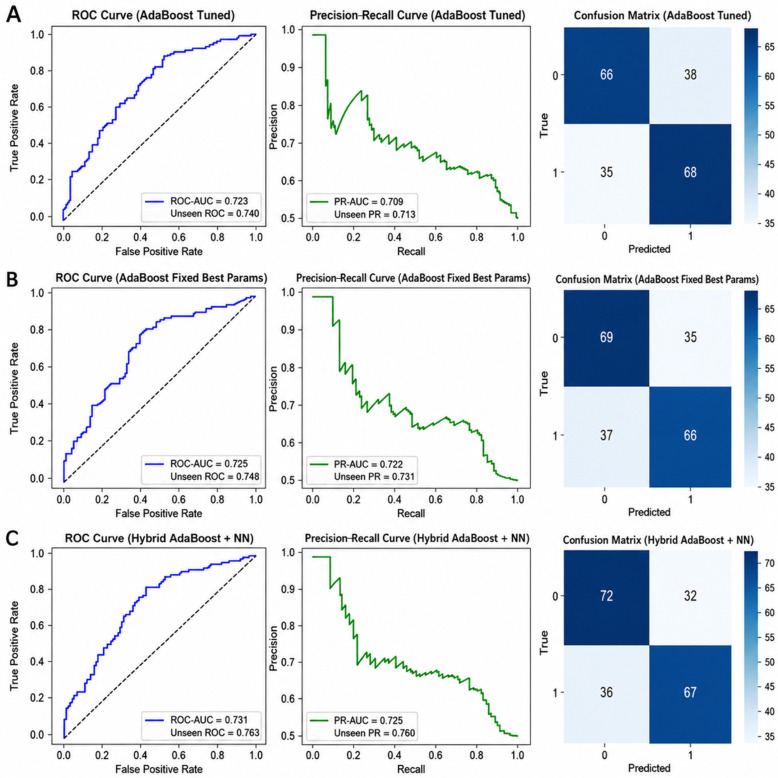
Performance comparison of AdaBoost-based models for Dataset-2. ROC curves, precision–recall curves, and confusion matrices for (**A**) tuned AdaBoost, (**B**) AdaBoost with fixed optimal hyperparameters, and (**C**) hybrid AdaBoost-NN model, demonstrating progressive gains in discrimination and stability.

Integrating the optimized AdaBoost with a neural-network component captured nonlinear dependencies and gave incremental gains (F1 = 0.663; ROC-AUC = 0.731), reflecting enhanced calibration and minority-class detection. The neural component contributed modestly (w_NN_ = 0.215), complementing the structural robustness of AdaBoost without inducing instability. Performance curves and confusion matrices for the baseline, tuned, and hybrid models are presented in Figure 4 (Panels A–C) and the values form all models were summarized in the Supplementary Table S6.

The modest absolute performance on Dataset 2 reflects its small size (1034 records) rather than a limitation of the balancing strategy; with only 517 positive cases shared between training and evaluation, estimates are necessarily less stable. This dataset was included to verify that the modelling pipeline behaves consistently under balanced conditions, and its results should be interpreted as a robustness check rather than as the primary model.

### 3.3. Model Development and Evaluation for Dataset-3

#### 3.3.1. Baseline Model Benchmarking

**Figure 5:**
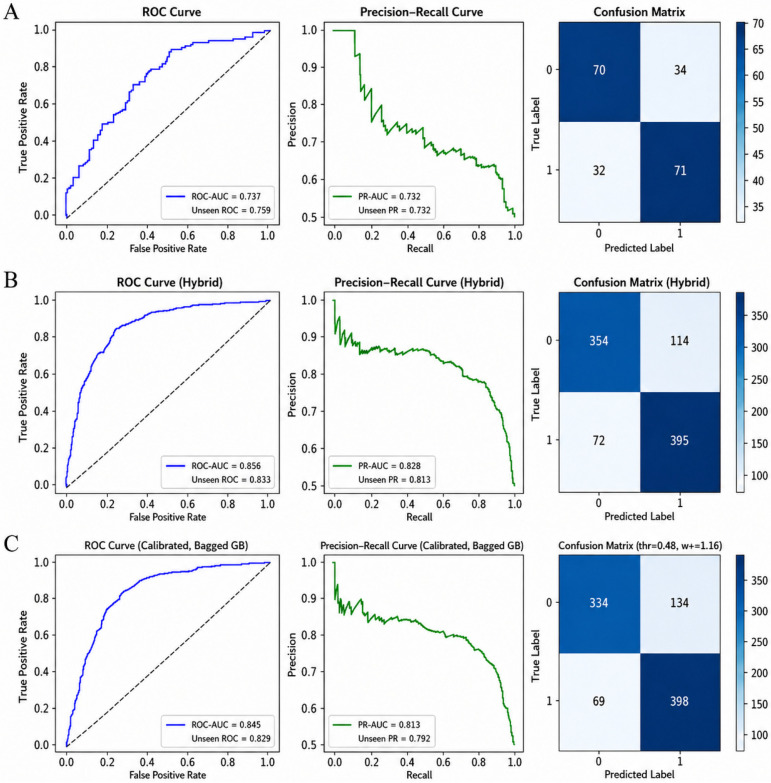
Comparative performance of three models for Dataset-3, including ROC curves, precision–recall curves, and confusion matrices for the Optuna-tuned Gradient Boosting model (**A**), the hybrid Gradient Boosting + Neural Network model (**B**), and the calibrated seed-bagged Gradient Boosting ensemble (**C**).

For Dataset-3, a systematic benchmarking procedure was conducted to identify the most suitable baseline learner for downstream optimization. Eight machine-learning classifiers, Logistic Regression, K-Nearest Neighbors, Support Vector Classifiers (linear and RBF kernels), Decision Tree, Random Forest, AdaBoost, Gradient Boosting (GB), and XGBoost, were evaluated under a unified preprocessing pipeline consisting of median imputation, z-score normalization, and synthetic minority oversampling (SMOTE) [27]. Data were partitioned using a 70%/20%/10% stratified split for training, testing, and unseen validation subsets to preserve class balance and ensure robust assessment of generalization. For Dataset-3 (*n* = 4672), this corresponded to 3270 training, 934 testing and 468 held-out “unseen” records; the “unseen” subset was used exclusively for a final generalization check and was never involved in model fitting or hyperparameter selection. This three-way partition is therefore distinct from the two-way 80:20 split used for the final deployed model (Section 2.6), and it is the source of the separate “Test” and “Unseen” values reported for the models in Figure 3, Figure 4 and Figure 5 and Table 3, Table 4 and Table 5.

Gradient Boosting showed the strongest discrimination and precision–recall balance (highest unseen PR-AUC 0.802; F1 0.758). XGBoost (PR-AUC = 0.793) and Random Forest (PR-AUC = 0.775) also performed competitively but did not match GB’s stability across folds. In contrast, simpler linear and distance-based models showed markedly lower precision–recall behavior, and the Decision Tree baseline produced the weakest generalization, reinforcing the importance of boosting-based aggregation for structured clinical datasets with class imbalance. These findings established Gradient Boosting as the Dataset-3 baseline for subsequent optimization, hybrid ensemble development, and calibration (Supplementary Table S7).

#### 3.3.2. Optuna-Tuned Gradient Boosting Performance

To enhance the performance of the baseline gradient boosting model, we employed Bayesian hyperparameter optimization using the Optuna framework. The objective function was a weighted composite metric designed to balance classification performance and discrimination:

0.7 × F1 + 0.2 × ROC-AUC + 0.1 × PR-AUC

This formulation emphasized precision–recall balance while retaining sensitivity to global class separation. The optimization process explored key hyperparameters, including learning rate, number of estimators, maximum tree depth, and subsampling ratios. After 80 optimization trials, the procedure converged to the optimal configuration: learning_rate = 0.0448, n_estimators = 151, max_depth = 3, and subsample = 0.647. The resulting tuned model demonstrated substantial improvements over the baseline, achieving the evaluation metrics in Table 3.

These results indicate improved generalization and discriminative performance relative to the baseline gradient boosting model (which yielded a test PR-AUC of 0.784), with minimal signs of overfitting. The model’s behavior and class separation capabilities are further illustrated in Figure 5A, which presents the ROC curve, precision–recall curve, and confusion matrix for visual reference.

#### 3.3.3. Hybrid Gradient Boosting and Neural Network Ensemble

To further augment predictive performance, a hybrid ensemble was constructed by integrating the optimized GB model with a DNN, leveraging the complementary strengths of tree-based and deep learning architectures. The DNN architecture consisted of three fully connected hidden layers (128, 64, and 32 neurons), employing ReLU activations, batch normalization after the first two layers, dropout regularization (rate = 0.3), and a sigmoid-activated output layer. The model was trained using the Adam optimizer (learning rate = 0.001) and binary cross-entropy loss, with early stopping (patience = 8) to prevent overfitting.

Ensemble blending was optimized using the Optuna framework, which searched for the optimal weighting of prediction probabilities from the two base learners. The final hybrid model assigned a dominant weight to the DNN component (w_nn_ = 0.705), indicating a 70.5% contribution from the neural network and 29.5% from the Gradient Boosting model. This distribution underscores the enhanced predictive capacity provided by non-linear deep learning representations in the ensemble output. The hybrid model achieved superior performance across all evaluation metrics as in Table 4.

Combining tree-based decision boundaries with DNN-derived feature abstractions yielded a more discriminative model: as shown in Figure 5B, the hybrid ensemble improved precision–recall behavior and maintained generalization on unseen data, the best-performing configuration across all tested approaches.

#### 3.3.4. Calibrated Seed-Bagged Gradient Boosting (CSB-GB)

To enhance probabilistic reliability and reduce model variance, a Calibrated Seed-Bagged Gradient Boosting (CSB-GB) ensemble was developed. This approach aimed to improve both predictive consistency and output calibration—key requirements for clinical decision-support systems. The ensemble consisted of five independently trained Gradient Boosting models, each initialized with a different random seed but sharing an identical optimized configuration (learning rate = 0.1, n_estimators = 300, max_depth = 3, subsample = 0.9). The final prediction was derived by averaging the class probabilities generated by each model, thereby mitigating randomness-induced variance and stabilizing ensemble outputs.

To ensure accurate probability estimates, each model underwent post hoc calibration using isotonic regression with five-fold cross-validation via the CalibratedClassifierCV method in scikit-learn. This step adjusted the raw prediction probabilities to better align with observed event rates, improving clinical interpretability. Further refinement was achieved through Bayesian optimization (Optuna), which fine-tuned two critical meta-parameters: the positive-class weighting factor (*pos_weight* = 1.159) and the classification decision threshold (τ = 0.484). These values optimized the trade-off between sensitivity and precision, as quantified by a multi-objective scoring function prioritizing F1-score, MCC, ROC-AUC, and PR-AUC. The final CSB-GB model yielded the results in Table 5.

Although marginally lower in peak discriminative performance than the hybrid ensemble, CSB-GB demonstrated the most consistent output across random seeds and superior calibration reliability. This robustness is particularly valuable in clinical applications where precise risk stratification is essential. As illustrated in Figure 5C, the model achieved a well-balanced trade-off between classification performance and probability accuracy, supporting its adoption in healthcare settings that prioritize actionable and interpretable risk predictions.

### 3.4. Model Explainability and SHAP-Based Interpretation

To provide clinically transparent and interpretable CVD risk predictions, model explainability was performed using SHapley Additive exPlanations (SHAP), a game-theoretic framework that quantifies each feature’s marginal contribution to prediction probability at both global and individual levels [39]. Although the hybrid Gradient Boosting and Neural Network ensemble achieved the highest discrimination performance, and the Optuna-tuned Gradient Boosting model delivered strong generalization, the Calibrated Seed-Bagged Gradient Boosting model was selected for interpretability analysis due to its well-calibrated probability outputs and greater clinical applicability for patient-level cardiovascular risk stratification. Probabilistic outputs are essential in clinical decision-support systems because they enable threshold-based interpretation and shared decision-making rather than providing only categorical outcomes [40,41]. Accordingly, SHAP summaries were generated for the CSB-GB model to examine the biological plausibility and clinical interpretability of predictions.

**Figure 6:**
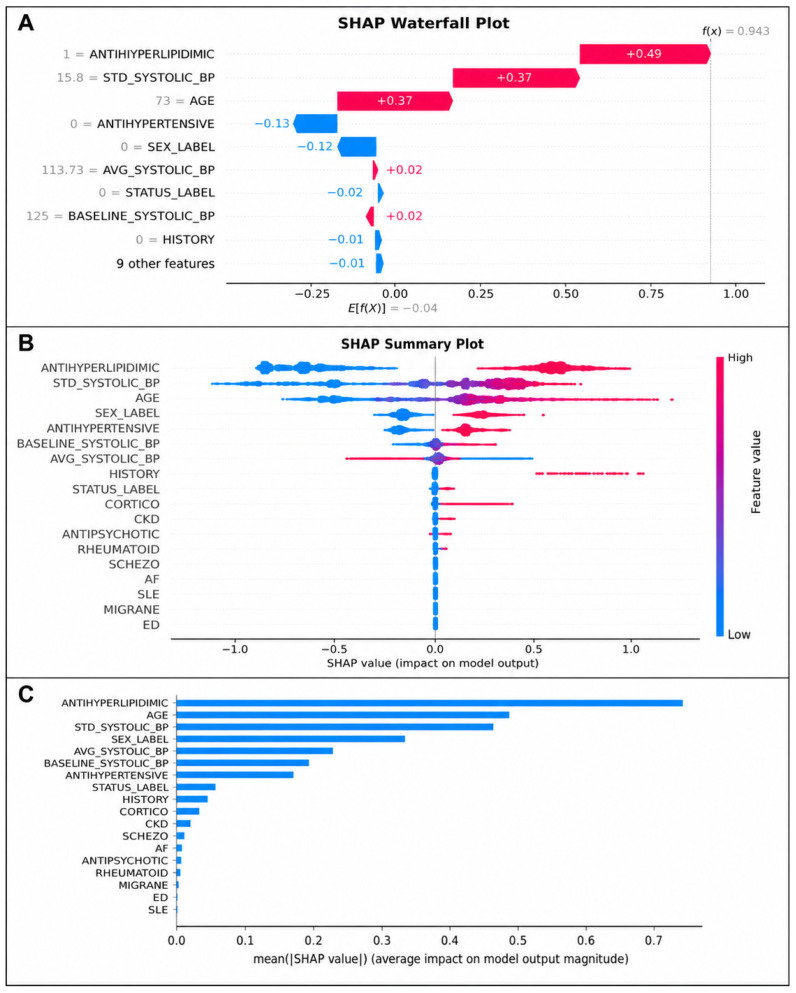
SHAP-based explanation of cardiovascular risk predictions. (**A**) SHAP waterfall plot illustrating individual-level feature contributions toward a high predicted cardiovascular disease risk probability. (**B**) SHAP summary plot showing global feature importance and directional impact across the cohort, highlighting lipid-lowering therapy use, systolic blood pressure variability, age, and sex as the most influential predictors. (**C**) Mean absolute SHAP value (global feature importance) for the deployed model, confirming antihyperlipidemic therapy, age, and systolic blood pressure variability as the dominant contributors.

The SHAP waterfall visualization (Figure 6A) illustrates the decomposition of an example patient’s predicted CVD probability, showing the additive influence of each clinical variable from the model baseline (E[f(x)] = −0.04) to the final estimated risk (f(x) = 0.943). Key positive contributors included antihyperlipidemic medication usage (+0.49), systolic blood pressure variability (STD_SYSTOLIC_BP, +0.37), and older age (+0.37), consistent with established epidemiological associations linking dyslipidemia, hemodynamic variability, and aging with increased cardiovascular risk [42,43]. Conversely, the absence of prior antihypertensive exposure (−0.13) and female sex (−0.12) reduced estimated risk, aligning with known sex-based cardiovascular protection in pre-menopausal women and documented higher CVD events in untreated hypertensive populations [21,44]. Additional parameters, including mean and baseline systolic blood pressure, demonstrated smaller incremental contributions (±0.02), highlighting the dominating importance of medication-related and variability-based predictors in individualized risk estimation.

At the population level, the SHAP summary plot (Figure 6B) demonstrated that antihyperlipidemic therapy was the most influential global feature, followed by systolic blood pressure variability, age, and sex. Higher STD_SYSTOLIC_BP and older age consistently yielded positive SHAP values, indicating elevated risk contributions, whereas female sex and antihypertensive therapy exposure exhibited protective effects. Comorbid inflammatory conditions such as chronic kidney disease, rheumatoid arthritis, and systemic lupus erythematosus displayed modest but directionally appropriate contributions, reflecting their recognized associations with chronic vascular injury and accelerated atherosclerosis [45,46].

#### Independent Reproduction and Advanced-Model Comparison

To verify reproducibility and to address whether a more complex learner would outperform the deployed model, the final pipeline was re-executed on an independent machine and benchmarked against a gradient-boosted decision-tree model (XGBoost) trained on the identical 80:20 split. The reproduction confirmed the reported performance of the calibrated seed-bagged Gradient Boosting model (CSB-GB/JoRisk), with a test ROC-AUC of 0.848, PR-AUC of 0.825, F1-score of 0.798, Matthew’s correlation coefficient (MCC) of 0.575 and a Brier score of 0.157, closely matching the values reported in Table 5. The XGBoost comparator achieved a marginally higher ROC-AUC (0.850) but a lower PR-AUC (0.818), the metric most relevant under class imbalance, together with a comparable F1-score (0.797), MCC (0.582) and Brier score (0.155) (Table 6).

These results show that a heavier, more flexible model does not yield a clinically meaningful improvement over the calibrated ensemble: the differences across all discrimination, classification and calibration metrics are within the range expected from random seeding. The calibrated seed-bagged Gradient Boosting model was therefore retained as the final model, since it matched the advanced learner on discrimination while providing superior probability calibration and transparent, SHAP-based interpretability; properties that are more important than marginal gains in raw discrimination for a clinical decision-support tool.

The bootstrap optimism correction provided a formal, whole-sample assessment of overfitting. Across 200 resamples, the mean optimism in discrimination was small (ROC-AUC optimism 0.054, 95% CI 0.046–0.062; PR-AUC optimism 0.070, 95% CI 0.060–0.082), yielding an optimism-corrected ROC-AUC of 0.861 and PR-AUC of 0.852; the optimism-corrected Brier score was 0.163 (Table 7). The optimism-corrected discrimination is consistent with, and marginally higher than, the held-out test estimate (ROC-AUC 0.844), indicating that the reported performance is not inflated by overfitting and that the held-out figures are, if anything, slightly conservative. Together with the close test-versus-unseen agreement in Figure 3, Figure 4 and Figure 5, this constitutes rigorous internal validation; it does not replace independent external validation, which is discussed below as the principal direction for future work.

### 3.5. Data Quality Limitations and Mitigation Strategies in the Jordanian EHR Cohort

Several data constraints in the Jordanian EHR affected both cohort construction and modeling. Foremost was the scarcity and inconsistency of clinical data: essential variables such as blood pressure, HbA1c, and lipid profiles (triglycerides, LDL, HDL, total cholesterol) were frequently missing or recorded in non-standard formats. To address these limitations, extensive preprocessing and harmonization were conducted using Python scripts, including unit standardization, error filtering, and plausibility checks [47] (e.g., excluding implausible values such as HbA1c > 20%). Repeated values were merged across timepoints to consolidate fragmented records. These steps reduced sample size substantially: from approximately 600,000 patients, 4672 met the inclusion criteria for Dataset-3, the final cohort for training and evaluation.

Several known risk modifiers were also absent: smoking status, body mass index (frequent missing height/weight), and socioeconomic indicators were not systematically available, and ICD-10 coding inconsistencies limited comorbidity-label reliability. Measurements were not consistently aligned across visits, and some values were recorded in multiple units (e.g., mg/dL and mmol/L) without annotation, necessitating rigorous normalization.

Importantly, the temporal coverage of the dataset spanned only five years (2018–2022), which constrained the model’s ability to learn long-term cardiovascular trajectories and limited outcome ascertainment to events occurring within this window. As a result, the CSB-GB model was inherently trained to predict near- to medium-term cardiovascular risk (within a 5-year horizon), rather than traditional 10-year projections commonly used in tools like QRISK3 or Framingham [10,47].

Despite these challenges, missingness was mitigated through three tiered datasets of varying feature completeness, allowing iterative robustness checks under different data-availability conditions. Our findings show that a model trained on high-integrity core variables can deliver clinically meaningful predictions even without certain features, underscoring that data quality, rather than feature quantity, is often the limiting factor in real-world healthcare ML. The final Calibrated Seed-Bagged Gradient Boosting (CSB-GB) model (‘JoRisk’) was implemented using Python and deployed via FastAPI. The model file and deployment scripts are publicly available at: https://github.com/Saeedmomo/Jordan_CVD_risk/blob/main/Calibrated_Bagged_GB_Model.pkl for reproducibility and external validation.

### 3.6. Comparison with a Conventional Risk-Factor Model and Clinical Net Benefit

**Figure 7:**
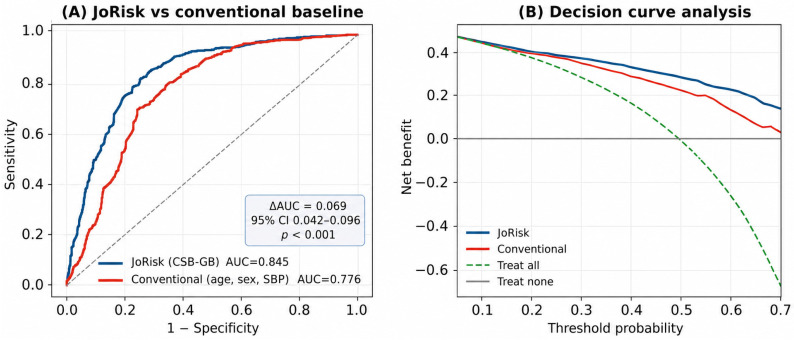
Added value and clinical utility of JoRisk (**A**) Receiver operating characteristic comparison of JoRisk and the conventional age/sex/blood pressure model on the same Dataset 3 test set. (**B**) Decision curve (net benefit) analysis across decision thresholds.

On the identical Dataset 3 test partition, the conventional model using only age, sex and baseline systolic blood pressure achieved an area under the curve of 0.776, whereas the calibrated seed-bagged Gradient Boosting model (JoRisk) achieved 0.845 (Figure 7A). The paired bootstrap confirmed that this improvement was statistically significant, with a difference in area under the curve of 0.069 (95% confidence interval 0.042 to 0.096; *p* < 0.001) and an increase in precision–recall area under the curve from 0.714 to 0.813. Benchmarked against published population-scale machine-learning models, JoRisk is competitive or superior: Weng et al. reported a best area under the curve of 0.745–0.764 on 378,256 UK records [18], and Alaa et al. reported 0.774 on 423,604 UK Biobank participants [20]. Accuracy figures of approximately 85% occasionally cited elsewhere typically derive from small, curated and artificially balanced benchmark datasets and report accuracy rather than area under the curve for incident events; they are therefore not directly comparable to discrimination measured on a national, prospectively defined incident disease cohort.

Decision curve analysis (Figure 7B) showed that JoRisk yielded higher net clinical benefit than both the treat-all and treat-none strategies and then the conventional model across the clinically relevant threshold range (for example, a net benefit of 0.372 versus 0.348 for the conventional model and 0.285 for treat-all at a threshold probability of 0.30), supporting its potential clinical usefulness. A formal cost-effectiveness and quality-adjusted-life-year evaluation will require linked cost and utility data and is planned as future work.

### 3.7. Subgroup Fairness and Algorithmic-Bias Assessment

**Figure 8:**
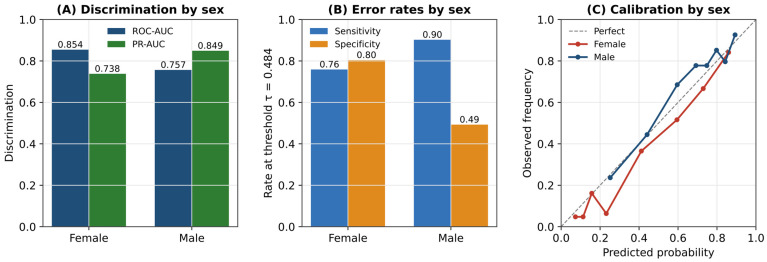
Sex-stratified performance of JoRisk on Dataset 3 test set, showing discrimination, error rates at the operating threshold, and calibration for female and male patients.

Discrimination was good in both sexes but differed in magnitude: the area under the curve was 0.854 in women and 0.757 in men. At the fixed decision threshold, error rates were unequal (sensitivity and specificity of 0.76 and 0.80 in women versus 0.90 and 0.49 in men), indicating that the model tends to over-flag male patients as high risk (Figure 8). We report this disparity transparently as a limitation. Practical mitigations include sex-specific decision thresholds, group-wise recalibration and ongoing performance monitoring after deployment. Calibration was acceptable in both groups, supporting the use of the calibrated probabilities for threshold-based decisions within each subgroup.

### 3.8. Data Governance, Privacy and Ethical Considerations

All analyses used only de-identified records, accessed under institutional and Ministry of Health approvals, with deduplication and de-identification performed within the Hakeem platform before extraction. No direct identifiers were available to the analysts, and no attempt was made to re-identify individuals. Because socioeconomic status and ethnicity are not recorded in the source system, fairness could be audited only with respect to sex; this is acknowledged as a limitation, and broader subgroup auditing is identified as a target for future linked-data studies. Consistent with guidance on algorithmic bias in healthcare [35], we recommend that any prospective deployment be accompanied by prespecified subgroup performance monitoring and a governance process for periodic recalibration.

## 4. Discussion

### 4.1. Model Performance Across Datasets and Robustness of Dataset 3

The three analytical datasets differed substantially in sample size, class balance and feature completeness, which directly influenced model performance and generalizability. Dataset 1 included the largest number of records with laboratory variables such as HbA1c and lipid measures, but its severe class imbalance, with approximately 5% CVD prevalence, limited minority-class detection. Although the linear support vector classifier was the strongest baseline model, its precision–recall performance remained modest. Subsequent Optuna tuning and neural-network integration improved discrimination, but the model remained constrained by imbalance and sparse outcome representation.

Dataset 2 was intentionally balanced but substantially smaller, comprising 1035 patients. In this setting, ensemble models performed best, with AdaBoost achieving the strongest baseline F1-score and PR-AUC. Further Bayesian optimisation and hybrid AdaBoost–neural network modelling produced modest improvements, supporting the value of balancing for minority-class detection. However, the reduced sample size limited the stability and generalizability of this dataset.

Dataset 3 provided the most robust modelling framework. By excluding sparsely available laboratory features and retaining widely captured variables, including age, sex, systolic blood pressure measures, comorbidities and medication exposure, this dataset expanded the usable cohort to 4672 records with balanced outcome classes. Despite a smaller feature set, Gradient Boosting achieved strong baseline performance, with unseen PR-AUC of 0.802 and F1-score of 0.758. After Bayesian optimisation, performance further improved, reaching test ROC-AUC of 0.855 and PR-AUC of 0.823, with preserved generalization on unseen data. These findings indicate that, in real-world EHR settings, feature completeness and sample representativeness may be more important than feature quantity. Dataset 3 therefore formed the most reliable basis for downstream model optimisation and clinical interpretation.

Rather than being redundant, the three datasets are integral to the central argument of this study and are retained deliberately. Dataset 1 demonstrates that a large cohort is, on its own, insufficient: despite the greatest number of records and the richest laboratory features, its severe class imbalance (approximately 5% prevalence) yields poor minority-class detection, illustrating that volume cannot compensate for imbalance. Dataset 2 demonstrates the opposite failure mode: although it is balanced and feature complete, with the laboratory measures typically considered essential for cardiovascular risk models, its small size limits stability and generalizability, showing that a feature-rich but small dataset is of limited practical value. Dataset 3 is the resolution of this tension, it combines the two advantages, achieving both adequate sample size and balanced outcomes by relying on routinely captured, high-completeness predictors. This progression from a large-but-imbalanced cohort, through a balanced-but-small cohort, to a balanced and adequately powered cohort built on routinely available data is itself a principal contribution of the work; removing Datasets 1 and 2 would obscure the rationale for Dataset 3 and the broader methodological lesson that, in real-world electronic health records, the optimal dataset is defined by the balance of size, class distribution and feature availability rather than by any one of these factors alone.

### 4.2. Clinical Relevance of the Calibrated Seed-Bagged Gradient Boosting Model

Within Dataset 3, the calibrated seed-bagged Gradient Boosting model was the most clinically suitable: although the hybrid GB–neural-network ensemble achieved marginally higher discrimination, the calibrated model offered a better balance of accuracy, calibration, reproducibility and interpretability. It averaged five independently seeded GB learners and applied isotonic calibration to align predicted probabilities with observed event rates.

The final model achieved strong discrimination (test ROC-AUC 0.844, PR-AUC 0.813) and maintained performance on unseen data, comparable to established risk-prediction tools and ML models in the literature [18,48]. Importantly, the model was optimised not only for discrimination but also for calibrated probability estimation. This distinction is clinically important because cardiovascular risk prediction is most useful when probabilities can support threshold-based decisions, preventive counselling, medication review and shared clinical decision-making.

An independent re-execution of the full pipeline reproduced these results (test ROC-AUC 0.848, PR-AUC 0.825), and a more complex gradient-boosted model (XGBoost) trained on the identical split did not outperform the calibrated ensemble in any clinically relevant respect (Section Independent Reproduction and Advanced-Model Comparison). This indicates that the compact, routinely available feature set is already exploited efficiently and that additional model complexity offers no meaningful benefit, reinforcing the choice of a transparent, well-calibrated model for clinical deployment.

Calibrated outputs make the model more suitable for decision support than an uncalibrated binary classifier, enabling stratification into interpretable risk categories relevant to interventions such as lifestyle modification, lipid-lowering therapy or antihypertensive intensification. The calibrated seed-bagged model thus balances predictive performance and clinical usability.

### 4.3. Data Completeness, Feature Selection and Interpretability

The results highlight an important methodological issue in real-world EHR-based modelling: more features do not necessarily yield better models when those features are sparsely or inconsistently recorded. Laboratory variables such as HbA1c and cholesterol are clinically relevant cardiovascular risk markers [49,50], but incomplete recording can reduce effective sample size, introduce selection bias and weaken generalizability. Dataset 1 illustrated this limitation, as the inclusion of laboratory features did not compensate for severe imbalance and missingness. In contrast, Dataset 3 achieved stronger performance by relying on more consistently available predictors, including blood pressure variability and medication exposure.

Thus medication records and longitudinal blood-pressure summaries can serve as clinically meaningful EHR-derived proxies: antihyperlipidemic use may reflect underlying dyslipidemia or elevated baseline risk, and systolic blood-pressure variability may capture unstable vascular control beyond a single measurement. Though not substitutes for laboratory testing, they improved transportability within a data-constrained national EHR. This aligns with previous evidence that machine learning models can improve cardiovascular risk prediction by exploiting routinely captured clinical patterns and nonlinear relationships not fully represented in conventional risk equations [18].

Interpretability was central to model selection. SHAP analysis was applied to the calibrated seed-bagged model to explain both individual and global predictions [39]. The most influential predictors were clinically plausible: antihyperlipidemic therapy, systolic blood pressure variability, age and sex. These factors are consistent with established cardiovascular epidemiology, including the roles of dyslipidemia, haemodynamic instability and ageing in cardiovascular risk [51,52]. Comorbid inflammatory and renal conditions, including chronic kidney disease, rheumatoid arthritis and systemic lupus erythematosus, showed smaller but directionally appropriate contributions, consistent with their recognised vascular risk associations. Compared with the hybrid neural model, the calibrated Gradient Boosting ensemble therefore provided a more transparent and clinically defensible modelling strategy, despite a small reduction in peak discrimination.

### 4.4. Comparison with Established Risk Tools, Limitations and Clinical Translation

Traditional scores such as QRISK3, Framingham and the ACC/AHA Pooled Cohort Equations remain widely used for their simplicity, interpretability and external validation in large populations [10]. QRISK3, for example, was developed using millions of UK patients and provides long-term cardiovascular risk estimates using Cox proportional hazards modelling. By contrast, the present model was developed from a smaller Jordanian EHR cohort and was designed for shorter-term event prediction within a five-year data window. Direct comparison with 10-year risk calculators should therefore be made cautiously.

Nevertheless, the model showed competitive discrimination and offered several practical advantages. First, it was trained on local EHR data, which may reduce the calibration drift that occurs when Western-derived risk scores are applied to non-Western populations. Second, it incorporated EHR-specific predictors such as treatment exposure and blood pressure variability, which may better reflect real-world clinical management. Third, isotonic calibration allowed risk probabilities to be aligned with observed local outcomes, strengthening its potential use as a context-specific decision-support tool. These findings support the broader view that machine-learning approaches can complement, rather than replace, established cardiovascular risk models by adapting prediction to local population structure and healthcare practice [18,53].

Several limitations remain. The model requires external validation in independent Jordanian and regional cohorts before deployment. Important predictors, including smoking status, body mass index, socioeconomic status and complete lipid/HbA1c profiles, were unavailable or inconsistently recorded, limiting direct comparison with conventional scores [54]. The five-year observation window also restricts the model to near- or medium-term prediction rather than long-term 10-year cardiovascular risk. In addition, although cross-validation and unseen testing reduced the likelihood of overfitting, machine-learning models may still capture site-specific patterns that do not generalize across health systems [55].

Three points warrant emphasis in light of these additional analyses. First, the available Hakeem records span only the 2018–2022 window; this period is too short to be partitioned into a separate, temporally independent external cohort while still retaining enough data to train and test the model reliably, since reserving a distinct time block for external testing would materially reduce the development sample and destabilise estimation. To guard against overfitting within this constraint, the benchmarking experiments used a three-way 70%/20%/10% stratified design in which the 10% “unseen” partition was withheld from all model fitting and hyperparameter selection; the close agreement between test and unseen performance indicates that the model is not overfit to the training cohort. We emphasise, however, that this held-out partition constitutes rigorous internal validation and is not a substitute for fully independent external validation across different time periods or healthcare systems, which remains the most important next step before clinical deployment. We therefore provide a ready-to-run validation protocol and frame the model’s generalizability claims accordingly. Second, the subgroup analysis demonstrated good discrimination in both sexes but unequal error rates, with a tendency to over-flag male patients; this fairness limitation, together with the mitigations described above, should be resolved before the tool informs individual care. Third, the decision-curve analysis indicated positive net benefit across the relevant threshold range, exceeding both default strategies and the conventional model, but a formal economic evaluation expressing benefit in quality-adjusted life-years will require linked cost and outcome data and is planned as future work.

## 5. Conclusions

This study introduces JoRisk, a clinically grounded machine learning-based model for predicting short-term CVD risk using real-world data from the Jordanian national electronic health record system. Developed on a large-scale cohort of over 600,000 patients and validated on carefully curated subsets, JoRisk demonstrated strong predictive performance even when faced with missing laboratory parameters; a common challenge in resource-limited clinical environments. The final model, a Calibrated Seed-Bagged Gradient Boosting ensemble, achieved high discrimination (ROC-AUC ≥ 0.84) and reliability (PR-AUC > 0.81), supported by robust cross-validation and external testing.

From a clinical standpoint, JoRisk offers several advantages. It incorporates variables directly available in routine care, such as age, sex, systolic blood pressure patterns, comorbidities, and medication history, enabling implementation without relying on complete lipid or glycemic profiles. This increases feasibility in low- and middle-income settings where laboratory testing may be inconsistent. The use of SHAP-based explainability confirms that the model’s predictions align with established pathophysiology, with risk contributions from blood pressure variability, age, chronic comorbidities, and pharmacologic exposures reflecting real-world cardiovascular risk.

By tailoring prediction to local data and avoiding dependence on imported Western equations, JoRisk addresses a gap in cardiometabolic prevention. It could inform earlier therapeutic decisions, prioritize high-risk individuals, and help reduce CVD-related morbidity and mortality in Jordan and comparable settings. Future steps include prospective validation and clinical integration.

## Figures and Tables

**Table 1:** Summary of the three constructed datasets, including selected variables, cardiovascular disease (CVD) prevalence, and total patient records, designed to assess the impact of missingness and feature selection on model robustness.

Dataset	Variables Included (Columns)	CVD Positives (%)	Total Records
Dataset 1	PATIENT_ID, AGE, SEX_LABEL, STATUS_LABEL, BASELINE_HBA1C, AVG_HBA1C, STD_HBA1C, HBA1C_LABEL, BASELINE_SYSTOLIC_BP, AVG_SYSTOLIC_BP, STD_SYSTOLIC_BP, CHOLESTEROL, HDL, CHOLES/HDL, TG, HISTORY, ED, AF, SLE, MIGRANE, CKD, RHEUMATOID, SCHIZO, ANTIPSYCHOTIC, ANTIHYPERLIPIDIMIC, ANTIHYPERTENSIVE, CORTICO, STD_HBA1C_missing, STD_SYSTOLIC_BP_missing	5%	10,130
Dataset 2	PATIENT_ID, AGE, SEX_LABEL, STATUS_LABEL, BASELINE_HBA1C, AVG_HBA1C, STD_HBA1C, HBA1C_LABEL, BASELINE_SYSTOLIC_BP, AVG_SYSTOLIC_BP, STD_SYSTOLIC_BP, CHOLESTEROL, HDL, CHOLES/HDL, TG, HISTORY, ED, AF, SLE, MIGRANE, CKD, RHEUMATOID, SCHIZO, ANTIPSYCHOTIC, ANTIHYPERLIPIDIMIC, ANTIHYPERTENSIVE, CORTICO, STD_HBA1C_missing, STD_SYSTOLIC_BP_missing	50%	1035
Dataset 3	PATIENT_ID, AGE, SEX_LABEL, STATUS_LABEL, BASELINE_SYSTOLIC_BP, AVG_SYSTOLIC_BP, STD_SYSTOLIC_BP, HISTORY, ED, AF, SLE, MIGRANE, CKD, RHEUMATOID, SCHIZO, ANTIPSYCHOTIC, ANTIHYPERLIPIDIMIC, ANTIHYPERTENSIVE, CORTICO	50%	4672

**Table 2:** Distribution of key demographic, clinical, and medication-related categorical variables stratified by cardiovascular disease (CVD) status, highlighting statistically significant associations with age, sex, comorbidities, and therapeutic exposures.

Variable	Category	CVD (%)	Non-CVD (%)	*p*-Value
Sex	Female	40.2	68.6	<0.001
	Male	59.8	31.4	
Age group	<40	13.0	15.1	<0.001
	40–60	40.8	37.4	
	>60	46.2	47.5	
Prior CVD	Yes	1.93	0.01	<0.001
Atrial fibrillation	Yes	1.76	0.34	<0.001
Chronic kidney disease	Yes	2.95	0.90	<0.001
Rheumatoid disease	Yes	0.73	0.68	1.00
Schizophrenia	Yes	1.97	0.86	0.002
Antipsychotic	Yes	2.20	1.14	<0.001
Antihyperlipidemic	Yes	49.6	47.3	<0.001
Antihypertensive	Yes	56.9	48.3	<0.001
Corticosteroids	Yes	6.02	5.04	<0.001

Values are presented as proportions (%). *p*-values derived from the Chi-square test (or Fisher’s exact test where cell counts < 5). A 95% confidence level (CL) was applied, and exact *p*-values are reported. Values below 0.001 are presented as *p* < 0.001.

**Table 3:** Performance of the Optuna-tuned Gradient Boosting model.

Metric	Test	Unseen
F1-Score	0.802	–
ROC-AUC	0.855	0.833
PR-AUC	0.823	0.809

**Table 4:** Performance of the hybrid Gradient Boosting–deep neural network ensemble.

Metric	Test	Unseen
F1-Score	0.809	–
ROC-AUC	0.857	0.833
PR-AUC	0.828	0.813

**Table 5:** Performance of the calibrated seed-bagged Gradient Boosting model.

Metric	Test	Unseen
F1-Score	0.797	–
ROC-AUC	0.844	0.822
PR-AUC	0.813	0.792

**Table 6:** Independent reproduction of the deployed model and comparison with an advanced gradient-boosted model (XGBoost) on the identical Dataset-3 test partition.

Metric (Test Set)	CSB-GB (JoRisk)	XGBoost
ROC-AUC	0.848	0.850
PR-AUC	0.825	0.818
F1-score	0.798	0.797
MCC	0.575	0.582
Brier score	0.157	0.155

**Table 7:** Bootstrap optimism-corrected internal validation of the deployed model (Harrell’s method, B = 200).

Metric	Apparent	Optimism	Corrected
ROC-AUC	0.915	0.054	0.861
PR-AUC	0.922	0.070	0.852
Brier (lower is better)	0.128	0.035	0.163

## Data Availability

The data presented in this study are openly available in GitHub at: https://github.com/Saeedmomo/Jordan_CVD_risk.

## Supplementary Materials

The following supporting information can be downloaded at: https://www.mdpi.com/article/10.3390/medsci14030343/s1, and https://github.com/Saeedmomo/Jordan_CVD_risk/blob/main/Calibrated_Bagged_GB_Model.pkl. Table S1: ICD-code–based definitions and inclusion criteria used to identify comorbid disease groups in the study cohort, including cardiometabolic, neurological, autoimmune, psychiatric, and cardiovascular conditions.; Table S2: Therapeutic medication classes and representative drugs included in the study, covering cardiovascular, metabolic, and neuropsychiatric treatments used for clinical feature extraction and risk modeling.; Table S3: Baseline performance comparison of machine learning classifiers for Dataset-1, reporting optimized hyperparameters and evaluation metrics across test and unseen validation sets; Table S4: Comparative performance of Optuna-tuned SVC, fixed SVC, and hybrid SVC–neural network models, evaluated using discrimination and calibration metrics on test and unseen datasets.; Table S5: Performance comparison of optimized machine learning classifiers for Dataset-2, showing tuned hyperparameters and predictive metrics across internal test and unseen validation cohorts.; Table S6: Performance comparison of Optuna-tuned AdaBoost, fixed-parameter AdaBoost, and hybrid AdaBoost–neural network models, evaluated using classification and discrimination metrics on test and unseen validation datasets.; Table S7: Performance comparison of optimized machine learning classifiers for Dataset-3, detailing selected hyperparameters and classification metrics across test and external unseen validation cohorts.

## References

[1] Roth G.A., Mensah G.A., Johnson C.O., Addolorato G., Ammirati E., Baddour L.M., Barengo N.C., Beaton A.Z., Benjamin E.J., Benziger C.P. (2020). Global burden of cardiovascular diseases and risk factors, 1990–2019: Update from the GBD 2019 study. J. Am. Coll. Cardiol..

[2] World Health Organization (2021). Cardiovascular Diseases (CVDs) Fact Sheet.

[3] Raman P., Sagadevan Y., Dhanapalan S., Fernandez B.J., Tan S.Y., Appalasamy J.R., Ramadas A. (2024). Lifestyle-related risk factors and primary prevention strategies for cardiovascular diseases in a middle-income country: A scoping review and implication for future research. J. Prev..

[4] Masaebi F., Salehi M., Kazemi M., Vahabi N., Azizmohammad Looha M., Zayeri F. (2021). Trend analysis of disability adjusted life years due to cardiovascular diseases: Results from the global burden of disease study 2019. BMC Public Health.

[5] Joseph P., Leong D., McKee M., Anand S.S., Schwalm J.-D., Teo K., Mente A., Yusuf S. (2017). Reducing the global burden of cardiovascular disease, part 1: The epidemiology and risk factors. Circ. Res..

[6] Leong D.P., Joseph P.G., McKee M., Anand S.S., Teo K.K., Schwalm J.-D., Yusuf S. (2017). Reducing the global burden of cardiovascular disease, part 2: Prevention and treatment of cardiovascular disease. Circ. Res..

[7] Chua H.M., Moshawih S., Kifli N., Goh H.P., Ming L.C., Goh K.W. (2025). Integrated Virtual Screening of Anthraquinone Derivatives for Anticancer Drug Discovery. J. Comput. Biophys. Chem..

[8] Cai Y., Cai Y.-Q., Tang L.-Y., Wang Y.-H., Gong M., Jing T.-C., Li H.-J., Li-Ling J., Hu W., Yin Z. (2024). Artificial intelligence in the risk prediction models of cardiovascular disease and development of an independent validation screening tool: A systematic review. BMC Med..

[9] D’Agostino R.B., Vasan R.S., Pencina M.J., Wolf P.A., Cobain M., Massaro J.M., Kannel W.B. (2008). General cardiovascular risk profile for use in primary care: The Framingham Heart Study. Circulation.

[10] Hippisley-Cox J., Coupland C., Brindle P. (2017). Development and validation of QRISK3 risk prediction algorithms to estimate future risk of cardiovascular disease: Prospective cohort study. BMJ.

[11] Goff D.C., Lloyd-Jones D.M., Bennett G., Coady S., D’agostino R.B., Gibbons R., Greenland P., Lackland D.T., Levy D., O’donnell C.J. (2014). 2013 ACC/AHA guideline on the assessment of cardiovascular risk: A report of the American College of Cardiology/American Heart Association Task Force on Practice Guidelines. J. Am. Coll. Cardiol..

[12] Logeswaran Y., Oliver D. (2025). It’s About Time: Why We Need to Consider Temporal Drift When Developing and Implementing Clinical Prediction Models. Biol. Psychiatry Cogn. Neurosci. Neuroimaging.

[13] Usher-Smith J.A., Silarova B., Schuit E., Moons K.G., Griffin S.J. (2015). Impact of provision of cardiovascular disease risk estimates to healthcare professionals and patients: A systematic review. BMJ Open.

[14] Khan S.U., Khan M.U., Virani S.S., Khan M.S., Khan M.Z., Rashid M., Kalra A., Alkhouli M., Blaha M.J., Blumenthal R.S. (2021). Efficacy and safety for the achievement of guideline-recommended lower low-density lipoprotein cholesterol levels: A systematic review and meta-analysis. Eur. J. Prev. Cardiol..

[15] Tsai M.L., Chen K.F., Chen P.C. (2025). Harnessing electronic health records and artificial intelligence for enhanced cardiovascular risk prediction: A comprehensive review. J. Am. Heart Assoc..

[16] Rane N.L., Paramesha M., Choudhary S.P., Rane J. (2024). Machine learning and deep learning for big data analytics: A review of methods and applications. Partn. Univers. Int. Innov. J..

[17] Akash O., raja Alzabin L., AbdElminaam D.S., Hesham Y., Hatem Y., Elserafy H., ElBahnesy K. (2025). From Data to Clinical Decision Support: Leveraging Feature Engineering and Machine Learning Techniques for Reliable Breast Cancer Detection and Diagnosis. Proceedings of 2025 International Mobile, Intelligent, and Ubiquitous Computing Conference (MIUCC).

[18] Weng S.F., Reps J., Kai J., Garibaldi J.M., Qureshi N. (2017). Can machine-learning improve cardiovascular risk prediction using routine clinical data?. PLoS ONE.

[19] Dey D., Slomka P.J., Leeson P., Comaniciu D., Shrestha S., Sengupta P.P., Marwick T.H. (2019). Artificial intelligence in cardiovascular imaging: JACC state-of-the-art review. J. Am. Coll. Cardiol..

[20] Alaa A.M., Bolton T., Di Angelantonio E., Rudd J.H., Van der Schaar M. (2019). Cardiovascular disease risk prediction using automated machine learning: A prospective study of 423,604 UK Biobank participants. PLoS ONE.

[21] Whelton P.K., Carey R.M., Aronow W.S., Casey D.E., Collins K.J., Dennison Himmelfarb C., DePalma S.M., Gidding S., Jamerson K.A., Jones D.W. (2018). 2017 ACC/AHA/AAPA/ABC/ACPM/AGS/APhA/ASH/ASPC/NMA/PCNA guideline for the prevention, detection, evaluation, and management of high blood pressure in adults: A report of the American College of Cardiology/American Heart Association Task Force on Clinical Practice Guidelines. J. Am. Coll. Cardiol..

[22] American Diabetes Association Professional Practice Committee (2024). 2. Diagnosis and classification of diabetes: Standards of care in diabetes—2024. Diabetes Care.

[23] Grundy S.M., Stone N.J., Bailey A.L., Beam C., Birtcher K., Blumenthal R. (2019). 2018 guideline on the management of blood cholesterol: A report of the American College of Cardiology/American Heart Association Task Force on Clinical Practice Guidelines. J. Am. Coll. Cardiol..

[24] Rice J.B., White A.G., Scarpati L.M., Wan G., Nelson W.W. (2017). Long-term systemic corticosteroid exposure: A systematic literature review. Clin. Ther..

[25] Chung Y.-S., Shao S.-C., Chi M.-H., Lin S.-J., Su C.-C., Kao Yang Y.-H., Yang Y.-K., Lai E.C.-C. (2021). Comparative cardiometabolic risk of antipsychotics in children, adolescents and young adults. Eur. Child Adolesc. Psychiatry.

[26] Albersen M., Orabi H., Lue T.F. (2011). Evaluation and treatment of erectile dysfunction in the aging male: A mini-review. Gerontology.

[27] Moshawih S., Bu Z.H., Goh H.P., Kifli N., Lee L.H., Goh K.W., Ming L.C. (2024). Consensus holistic virtual screening for drug discovery: A novel machine learning model approach. J. Cheminformatics.

[28] Chawla N.V., Bowyer K.W., Hall L.O., Kegelmeyer W.P. (2002). SMOTE: Synthetic minority over-sampling technique. J. Artif. Intell. Res..

[29] Akiba T., Sano S., Yanase T., Ohta T., Koyama M. Optuna: A next-generation hyperparameter optimization framework. Proceedings of the 25th ACM SIGKDD International Conference on Knowledge Discovery & Data Mining.

[30] Pedregosa F., Varoquaux G., Gramfort A., Michel V., Thirion B., Grisel O., Blondel M., Prettenhofer P., Weiss R., Dubourg V. (2011). Scikit-learn: Machine learning in Python. J. Mach. Learn. Res..

[31] Ioffe S., Szegedy C. Batch normalization: Accelerating deep network training by reducing internal covariate shift. Proceedings of the International Conference on Machine Learning.

[32] Kinga D., Adam J.B. A method for stochastic optimization. Proceedings of the International Conference on Learning Representations (ICLR).

[33] Niculescu-Mizil A., Caruana R. Predicting good probabilities with supervised learning. Proceedings of the 22nd International Conference on Machine Learning.

[34] Vickers A.J., Elkin E.B. (2006). Decision curve analysis: A novel method for evaluating prediction models. Med. Decis. Mak..

[35] Obermeyer Z., Powers B., Vogeli C., Mullainathan S. (2019). Dissecting racial bias in an algorithm used to manage the health of populations. Science.

[36] Iba K., Shinozaki T., Maruo K., Noma H. (2021). Re-evaluation of the comparative effectiveness of bootstrap-based optimism correction methods in the development of multivariable clinical prediction models. BMC Med. Res. Methodol..

[37] Harrell F.E., Lee K.L., Mark D.B. (1996). Multivariable prognostic models: Issues in developing models, evaluating assumptions and adequacy, and measuring and reducing errors. Stat. Med..

[38] Collins G.S., Moons K.G., Dhiman P., Riley R.D., Beam A.L., Van Calster B., Ghassemi M., Liu X., Reitsma J.B., Van Smeden M. (2024). TRIPOD+ AI statement: Updated guidance for reporting clinical prediction models that use regression or machine learning methods. BMJ.

[39] Lundberg S.M., Lee S.-I. (2017). A unified approach to interpreting model predictions. Adv. Neural Inf. Process. Syst..

[40] Steyerberg E.W. (2019). Evaluation of clinical usefulness. Clinical Prediction Models: A Practical Approach to Development, Validation, and Updating.

[41] Collins G.S., Reitsma J.B., Altman D.G., Moons K.G. (2015). Transparent reporting of a multivariable prediction model for individual prognosis or diagnosis (TRIPOD): The TRIPOD statement. J. Br. Surg..

[42] Arnett D.K., Blumenthal R.S., Albert M.A., Buroker A.B., Goldberger Z.D., Hahn E.J., Himmelfarb C.D., Khera A., Lloyd-Jones D., McEvoy J.W. (2019). 2019 ACC/AHA guideline on the primary prevention of cardiovascular disease: A report of the American College of Cardiology/American Heart Association Task Force on Clinical Practice Guidelines. J. Am. Coll. Cardiol..

[43] American Diabetes Association (2022). Standards of medical care in diabetes—2022 abridged for primary care providers. Clin. Diabetes.

[44] Mosca L., Benjamin E.J., Berra K., Bezanson J.L., Dolor R.J., Lloyd-Jones D.M., Newby L.K., Piña I.L., Roger V.L., Shaw L.J. (2011). Effectiveness-based guidelines for the prevention of cardiovascular disease in women—2011 update: A guideline from the American Heart Association. Circulation.

[45] Conrad N., Verbeke G., Molenberghs G., Goetschalckx L., Callender T., Cambridge G., Mason J.C., Rahimi K., McMurray J.J., Verbakel J.Y. (2022). Autoimmune diseases and cardiovascular risk: A population-based study on 19 autoimmune diseases and 12 cardiovascular diseases in 22 million individuals in the UK. Lancet.

[46] Henein M.Y., Vancheri S., Longo G., Vancheri F. (2022). The role of inflammation in cardiovascular disease. Int. J. Mol. Sci..

[47] Jackson R., Marshall R., Kerr A., Riddell T., Wells S. (2009). QRISK or Framingham for predicting cardiovascular risk?. BMJ.

[48] Azoulay L.-D., Broussaud T., Kachenoura N., Mathian A., Pha M., Hié M., Ait Abdallah N., Pineton de Chambrun M., Papo M., Cohen-Aubart F. (2025). Performance of SCORE2, QRISK3 and PREVENT equations in systemic lupus erythematosus. Lupus.

[49] Borg R., Kuenen J., Carstensen B., Zheng H., Nathan D.M., Heine R.J., Nerup J., Borch-Johnsen K., Witte D., ADAG Study Group (2011). HbA1c and mean blood glucose show stronger associations with cardiovascular disease risk factors than do postprandial glycaemia or glucose variability in persons with diabetes: The A1C-Derived Average Glucose (ADAG) study. Diabetologia.

[50] DeFilippis A.P., Young R., Carrubba C.J., McEvoy J.W., Budoff M.J., Blumenthal R.S., Kronmal R.A., McClelland R.L., Nasir K., Blaha M.J. (2015). An analysis of calibration and discrimination among multiple cardiovascular risk scores in a modern multiethnic cohort. Ann. Intern. Med..

[51] Kostis J.B. (2007). The importance of managing hypertension and dyslipidemia to decrease cardiovascular disease. Cardiovasc. Drugs Ther..

[52] Lakatta E.G. (2002). Age-associated cardiovascular changes in health: Impact on cardiovascular disease in older persons. Heart Fail. Rev..

[53] Ma J., Zhang J., Li R., Zheng H., Li W. (2022). Using Bayesian optimization to automate the calibration of complex hydrological models: Framework and application. Environ. Model. Softw..

[54] Mansoor A.R., Abed A., Alqudah A., Alsayed A.R. (2025). Assessment of Medical Care Strategies for Primary Hypertension in Iraqi Adults: A Hospital-Based Problem-Oriented Plan. Patient Prefer. Adherence.

[55] Zubair M., Hussain M., Al-Bashrawi M.A., Bendechache M., Owais M. (2025). A comprehensive review of techniques, algorithms, advancements, challenges, and clinical applications of multi-modal medical image fusion for improved diagnosis. Comput. Methods Programs Biomed..

