# Supplementary material for: Real-World Pharmacotherapy-Driven Cardiovascular Risk Prediction Using Interpretable Machine Learning and Jordanian EHR Data
Source: Med Sci (Basel). 2026 Jun 24;14(3):343. doi: 10.3390/medsci14030343 (PMC13413450; doi:10.3390/medsci14030343)
Supplement: Supplementary file 1 [file medsci-14-00343-s001.zip › medsci-4317992-supplementary.pdf]

Supplementary Materials: Real-World Pharmacotherapy-Driven Cardiovascular Risk Prediction Using Interpretable Machine Learning and Jordanian EHR Data

Supplementary Table S1: ICD-code–based definitions and inclusion criteria used to identify comorbid disease groups in the study cohort, including cardiometabolic, neurological, autoimmune, psychiatric, and cardiovascular conditions.

Disease Group	Inclusion Diagnosis Criteria (according to ICD Codes)
**History**	Family history of ischemic heart disease, other circulatory diseases, disorders of lipoprotein metabolism, stroke, and sudden cardiac death; Personal history of transient ischemic attack (TIA) and cerebral infarction without residual deficits.
**Diabetes**	A comprehensive set of diagnoses including Type 1 and Type 2 diabetes mellitus with various complications (e.g., nephropathy, retinopathy, neuropathy, foot ulcers), drug-induced diabetes, prediabetes, and other specified forms.
**Depression**	Postpartum depression and unspecified depression.
**Cerebral Infarction**	Cerebral infarction due to embolism or stenosis, unspecified cerebral infarction, and sequelae such as ataxia, facial weakness, hemiplegia, and monoplegia.
**Migraine**	Various types including chronic migraine, menstrual migraine, migraine with and without aura (intractable and not intractable), and ophthalmoplegic migraine.
**Systemic Lupus Erythematosus (SLE)**	Discoid lupus, lupus anticoagulant syndrome, systemic lupus erythematosus (both specified and unspecified forms).
**Rheumatoid Arthritis (RA)**	Rheumatoid arthritis (with and without rheumatoid factor), rheumatoid nodules at specified sites (e.g., hand, hip, knee, elbow), rheumatoid myopathy, and bursitis.
**Chronic Kidney Disease (CKD)**	CKD stages 1-5, hypertensive CKD, and CKD associated with diabetes mellitus.
**Severe Mental Illness (SMI)**	Bipolar disorders, schizoaffective disorders, schizophrenia spectrum disorders, major depressive disorder (recurrent and severe), and obsessive-compulsive disorders.
**Cardiovascular Disease (CVD)**	Acute coronary syndromes, myocardial infarction, angina pectoris, coronary atherosclerosis, aortic atherosclerosis, transient ischemic attack (TIA), and other forms of acute ischemic heart disease.
**Atrial Fibrillation**	Paroxysmal, persistent, permanent, and chronic atrial fibrillation, as well as typical and atypical atrial flutter.

Supplementary Table S2: Therapeutic medication classes and representative drugs included in the study, covering cardiovascular, metabolic, and neuropsychiatric treatments used for clinical feature extraction and risk modeling.

Therapeutic Class	Medications
**Atypical Antipsychotics**	Clozapine, Olanzapine, Quetiapine, Risperidone
**Corticosteroids**	Prednisolone, Dexamethasone, Hydrocortisone
**Calcium Channel Blockers**	Verapamil, Diltiazem, Nifedipine, Amlodipine
**Vasodilator**	Hydralazine
**Diuretics**	Hydrochlorothiazide, Indapamide, Furosemide, Spironolactone, Amiloride
**ACE Inhibitors**	Captopril, Enalapril, Lisinopril, Perindopril, Ramipril
**Angiotensin II Receptor Blockers (ARBs)**	Losartan, Candesartan, Valsartan, Telmisartan
**Beta-Blockers**	Propranolol, Metoprolol, Atenolol, Carvedilol
**Alpha-Blocker / Central Agent**	Doxazosin, Methyldopa
**Phosphodiesterase-5 Inhibitors (Erectile Dysfunction)**	Sildenafil, Tadalafil
**Statins (Lipid-Lowering Agents)**	Atorvastatin, Fluvastatin, Pravastatin, Rosuvastatin, Simvastatin
**Fibrates (Lipid-Lowering Agents)**	Bezafibrate, Fenofibrate, Gemfibrozil
**Angiotensin Receptor-Neprilysin Inhibitor (ARNI)**	Sacubitril

Supplementary Table S3: Baseline performance comparison of machine learning classifiers for Dataset-1, reporting optimized hyperparameters and evaluation metrics across test and unseen validation sets.

Model	Best Params	Accuracy	Precision	Recall	F1-score	ROC-AUC	PR-AUC	Unseen ROC-AUC	Unseen PR-AUC
Logistic Regression	C = 10 (PCA = 15)	0.661	0.090	0.621	0.157	0.706	**0.142**	0.746	0.119
K-Nearest Neighbors	n_neighbors = 15 (PCA = 15)	0.659	0.069	0.456	0.120	0.609	0.074	0.688	0.096
SVC (Linear)	C = 0.1 (PCA = 15)	0.637	0.087	0.650	0.154	**0.711**	**0.143**	**0.749**	**0.122**
SVC (RBF)	C = 0.1, γ = 0.01 (PCA = 15)	0.629	0.088	0.670	0.155	0.701	0.105	0.737	0.117
Decision Tree	max_depth = 10 (PCA = 15)	0.688	0.086	0.534	0.148	0.649	0.085	0.658	0.083
Random Forest	max_depth = 5, n_estimators = 200 (PCA = 15)	0.710	0.091	0.524	0.155	0.685	0.098	0.716	0.108
Gradient Boosting	lr = 0.1, n_estimators = 50 (PCA = 15)	0.690	0.085	0.524	0.147	0.672	0.097	0.723	0.108
AdaBoost	lr = 0.1, n_estimators = 100 (PCA = 15)	0.628	0.073	0.544	0.129	0.656	0.082	0.686	0.092
XGBoost	lr = 0.1, max_depth = 3, n_estimators = 50 (PCA = 15)	0.220	0.060	**0.971**	0.112	0.662	0.102	0.710	0.102

Supplementary Table S4: Comparative performance of Optuna-tuned SVC, fixed SVC, and hybrid SVC–neural network models, evaluated using discrimination and calibration metrics on test and unseen datasets.

Metric	Optuna-Tuned SVC(C = 0.0638)	Fixed SVC(C = 0.0638)	Hybrid SVC + NN(C = 0.0638, wNN = 0.35)
**Accuracy**	0.950	0.950	0.950
**Precision**	1.000	1.000	1.000
**Recall**	0.019	0.019	0.019
**F1-score**	0.038	0.038	0.038
**MCC**	—	0.136	0.136
**ROC-AUC (Test)**	0.596	0.596	**0.719**
**PR-AUC (Test)**	0.090	0.090	**0.126**
**ROC-AUC (Unseen)**	0.634	0.634	**0.714**
**PR-AUC (Unseen)**	0.121	0.121	**0.155**

Supplementary Table S5: Performance comparison of optimized machine learning classifiers for Dataset-2, showing tuned hyperparameters and predictive metrics across internal test and unseen validation cohorts.

Model	Best Parameters	Accuracy	Precision	Recall	F1-score	ROC-AUC	PR-AUC	Unseen ROC-AUC	Unseen PR-AUC
Logistic Regression	C = 0.1	0.614	0.611	0.635	0.623	0.692	0.678	0.684	0.672
KNN	n_neighbors = 15	0.638	0.649	0.606	0.627	0.695	0.651	0.679	0.639
SVC (Linear)	C = 0.1	0.633	0.635	0.635	0.635	0.692	0.674	0.676	0.658
SVC (RBF)	C = 1, γ = 0.01	0.643	0.644	0.644	0.644	0.691	0.667	0.659	0.651
Decision Tree	max_depth = 10	0.551	0.552	0.558	0.555	0.562	0.537	0.579	0.549
Random Forest	max_depth = 5, n_estimators = 200	0.671	0.667	0.692	0.679	0.710	0.673	0.673	0.612
Gradient Boosting	learning_rate = 0.1, n_estimators = 50	0.671	0.673	0.673	0.673	0.709	0.675	0.639	0.602
**AdaBoost**	**learning_rate = 0.1, n_estimators = 100**	**0.662**	**0.655**	**0.692**	**0.673**	**0.716**	**0.714**	**0.680**	**0.643**
XGBoost	learning_rate = 0.1, max_depth = 3, n_estimators = 50	0.652	0.663	0.625	0.644	0.710	0.675	0.667	0.639

Supplementary Table S6: Performance comparison of Optuna-tuned AdaBoost, fixed-parameter AdaBoost, and hybrid AdaBoost–neural network models, evaluated using classification and discrimination metrics on test and unseen validation datasets.

Metric	Optuna-Tuned AdaBoost	Fixed Best-Params AdaBoost	Hybrid AdaBoost + NN
**Accuracy**	0.6473	0.6522	**0.6715**
**Precision**	0.6415	0.6535	**0.6768**
**Recall**	**0.6602**	0.6408	0.6505
**F1-score**	0.6507	0.6471	**0.6634**
**MCC**	0.2949	0.3043	**0.3431**
**ROC-AUC (Test)**	0.7230	0.7246	**0.7307**
**PR-AUC (Test)**	0.7094	**0.7225**	**0.7248**
**Unseen ROC-AUC**	**0.7400**	0.7483	0.763
**Unseen PR-AUC**	0.7134	**0.7310**	0.750

Supplementary Table S7: Performance comparison of optimized machine learning classifiers for Dataset-3, detailing selected hyperparameters and classification metrics across test and external unseen validation cohorts.

Model	Optimal Parameters	Accuracy	Precision	Recall	F1-score	ROC-AUC	PR-AUC	Unseen ROC-AUC	Unseen PR-AUC
Logistic Regression	C = 0.1	0.742	0.734	0.761	0.747	0.801	0.754	0.811	0.755
K-Nearest Neighbors	n_neighbors = 15	0.738	0.735	0.746	0.740	0.797	0.767	0.801	0.764
SVC (Linear)	C = 0.1	0.699	0.709	0.677	0.693	0.783	0.727	0.801	0.747
SVC (RBF)	C = 1, γ = 0.01	0.703	0.705	0.699	0.702	0.790	0.745	0.807	0.768
Decision Tree	max_depth = 10	0.694	0.705	0.669	0.686	0.711	0.669	0.732	0.668
Random Forest	n_estimators = 100, max_depth = 10	0.751	0.738	0.778	0.758	0.809	0.779	0.819	0.775
**Gradient Boosting**	**learning_rate = 0.1, n_estimators = 50**	**0.753**	**0.743**	**0.774**	**0.758**	**0.818**	**0.784**	**0.828**	**0.802**
AdaBoost	learning_rate = 0.1, n_estimators = 100	0.751	0.736	0.782	0.759	0.795	0.750	0.818	0.786
XGBoost	learning_rate = 0.1, max_depth = 3, n_estimators = 50	0.756	0.744	0.782	0.763	0.821	0.781	0.827	0.793
